# Our Early Experience in Surgical and Clinical Outcome on Endoscopic Cubital Tunnel Release: A Preliminary Result

**DOI:** 10.5402/2011/427403

**Published:** 2011-07-31

**Authors:** Philip Ian Barlaan, Josephine Wing-Yuk Ip

**Affiliations:** Division of Hand and Foot Surgery, Department of Orthopaedics and Traumatology, Hong Kong University Medical Centre, Queen Mary Hospital, 102 Pok Fu Lam Road, Hong Kong Island, Hong Kong

## Abstract

Cubital tunnel syndrome is one of the common upper extremity problem encountered. A mild syndrome can be often treated without surgery, but a failure of conservative treatment with constant symptoms or muscle atrophy and weakness requires surgical intervention. Despite the fact that is the second most common nerve entrapment in the upper limb, there is no accepted gold standard in the surgical management. But with the new technique in minimally invasive surgery and available endoscope, it addresses all potential compression sites with good visualisation but with small surgical exposure. The procedure is safe and reliable way to address this problem.

## 1. Introduction

Entrapment of the ulnar nerve in the elbow region is the second most common compression neuropathy in the upper extremity. There is no accepted standard for surgical treatment [1]. Many procedures have been advocated for decompression of the ulnar nerve at the elbow, including anterior subcutaneous transposition [2], anterior intramuscular transposition [3], anterior submuscular transposition [4], medial epicondylectomy [5], simple decompression [6], neurolysis [7], and in situ decompression. 

 The introduction of endoscopic release, the newest of all the surgical options for this problem has been described by several authors. The new approach to peripheral nerve surgery specifically in cubital tunnel syndrome is the introduction of the endoscopic procedure. Since the advent of endoscopic methods to release the course of the ulnar nerve entrapment at the elbow site, there has been a flurry of interest and controversy about the efficacy and safety of this newest technique.

 Endoscopic decompression of the ulnar nerve at the elbow was first described in 1995 by Tsai et al. [8]. Multiple variations of endoscopic technique have been described since then [9]. There are different variations of endoscopic surgical technique, but the purpose and goals preserve the vascularity of the ulnar nerve at the elbow, release all possible compression sites, allow early mobilisation of the elbow, avoid extensive surgical exposure, and scar discomfort. It provides for limited soft tissue dissection, thereby allowing more rapid recovery with minimal scarring [10].

 Potential ulnar nerve entrapment can occur at five sites around the elbow: the arcade of struthers, the medial intermuscular septum, the medial epicondyle, the cubital tunnel, and the deep flexor pronator aponeurosis. The most common site of entrapment is the cubital tunnel [11].

 Various surgical techniques for decompression of the ulnar nerve have been described in the literature, and a definitive gold standard does not exist [9]. Comparative studies have shown some short-term advantages to one or another technique, but overall results between the treatments have essentially been equivocal [9]. A thorough preoperative diagnosis and workup will help guide us for the type of surgical technique. We report our experience with this newer technique in six patients, with special focus on the clinical and surgical outcome.

## 2. Method

The study was carried out in the Department of Orthopaedics and Traumatology, Division of Hand and Foot Surgery, Hong Kong University Medical Centre at Queen Mary Hospital from period of February 2008 to May 2010. Their data was retrieved from Queen Mary Hospital and David trench Rehabilitation Centre. There were six cases with endoscopic cubital tunnel release. The Inclusion criteria are failure of conservative treatment for at least 6 months, have progressive clinical findings of atrophy, elevated two-point sensory discrimination, weakness of the ulnar nerve distribution, and positive electrophysiological conformation studies. The exclusion criteria include the subluxation of the ulnar nerve if it was felt to subluxate over the medial epicondyle on passive elbow flexion, long-standing elbow contracture, mass or space-occupying lesions, history of significant trauma of the elbow, history of cervical neuropathy or the double crush syndrome, prior surgery, scarred, adherent nerve, history of fracture of the elbow malunion, and cubital valgus.

 All patients underwent conservative management with splintage, avoidance of provocative activities, and rehabilitation program. Failure of conservative management and positive electrophysiological studies with clinical weakness of the ulnar nerve distribution and/or muscle atrophy is the criteria in surgical management. All six patients were offered endoscopic release as the alternative for standard open release procedure.

 A uniform endoscopic cubital tunnel release was done with a single senior orthopaedic hand surgeon with experience in endoscope release technique.

 Six patients who had compressive cubital tunnel syndrome at the elbow were treated with endoscopic CuTR at our institution after electrophysiological conformation of the diagnosis. The sensory conduction studies (i.e., amplitude of the sensory action potential and sensory conduction velocity of the ulnar nerve) were considered prolonged in all cases; the motor conduction studies (i.e., nerve site, onset, amplitude, segment, latency difference, distance, and conduction velocity) were prolonged in all cases. The Dellon's scale score was used for rating the severity of the lesions and the postoperative outcome was assessed based on the modified Bishop rating scale system.

The final postoperative outcome was assessed 6 months after the surgery by subjective information based on Modified Bishop Scoring Classification System (severity of residual system, improvement, work status, strength, and sensibility), this Bishop score is defined as poor, 0 to 2; fair, 3 to 4; good, 5 to 7; excellent, 8 to 9. The objective parameters (grip strength and sensory two-point discrimination). The mean followup postoperatively was 33 months (range, 9–33). The outcomes were evaluated clinically.

## 3. Surgical Technique

Surgery is performed in patient under GA in supine position with upper limb side table. The patient shoulder in abduction and external rotated and elbow slightly extended in position. A pneumatic tourniquet applied in the most proximal area of the arm, a clearly marked of anatomical portal and course of the ulnar nerve. A 1.5 cm–3 cm transverse incision is made between the medial humeral epicondyle and the olecranon at the course of the ulnar nerve. The ulnar nerve has been identified, subcutaneous pouch developed and endoscope set inserted (KARL STORZ, Tuttlingen, Germany) into subcutaneous tunnel and release under endoscopic guidance proximally. Another distal pouch developed with the same entry site, and endoscope has been inserted. A distal release into two heads of flexor carpi ulnaris and the nerve branches of the FCU have been identified and protected. The endoscope has been removed then elbow flexed and checked for ulnar nerve subluxation. Saline irrigation and drain was inserted and anchored in view of oozing from minor vessels and clot formation. Wound closure with nylon 5/0 monofilament suture. A soft dressing is applied with long-arm backslab in slightly 45 degrees elbow extension as haemostasis for two days, and gentle active mobilisation exercise. 

## 4. Result

Our patient population in this series is six patients: two woman and four men with 1 : 2 being the ratio. The median average age was 55 years (range, 33–77). A surgical endoscopic release was performed on the right side in four elbows and on the left side in two elbows. The main job profile of two patients is mainly table-top tasks, one works as dental hygienist, and three are nonworking in their retirement age, and all patients were not manual labourer. In five patients, the right side is dominant except one patient who was ambidextrous (Table 1).

The mean length of the surgery in the endoscopic release average 47 minutes (range, 32–62 minutes). The mean length of the skin incision was 2.25 cm (range, 1.5–3 cm). Retrospectively, no patient was classified as mild, three patients (50%) were moderate, and three (50%) were severe according to Dellon's classification in stages of ulnar nerve compression at the elbow (Table 2).

The postoperative outcome result is based on modified Bishop rating system classification based on severity of residual symptoms, improvement of symptoms, work status, strength, and sensitivity which shows two (33%) with excellent results, and four (66%) have good results (Table 3).

Three working group patients return to work with their previous job description. The dental hygienist who has major risk factors identified sustained exposed to work related repetitive left elbow flexion more than 90 degrees constantly during the therapy session (>1 hour) and sustained holding of hand tools. Surgical site is not the dominant hand, then exposure to the vibratory tooth scaler machine is not a risk factor. There is no change of job to this patient, but with recommendations incorporate half-hour sessions of duties avoiding sustained gripping or sustaining affected elbow flexion >90 degrees after every half-hour of therapy session and regular intermittent stretching of left elbow during the therapy session. This patient has excellent modified Bishop score. Three patients who are in retirement age with initial subjective evaluation base on modified Bishop's classification reach to score of good result without adding the subtype point score of work status of the patients for the reason, and this is not applicable to this group of patients (Table 4). 

None of the patient was converted endoscopic release to in situ open due to any complication. No patients develop a postoperative infection, cutaneous nerve injury, hematoma, or painful surgical site. All patients improved symptom one day after the surgery with sensory loss improved and go back to their full activity in one month, and three patients previously went back to work after two months. The recovery and return to work was rapid and with a high patient satisfaction and no recurrence of symptom noted. None of the patients complained about scar discomfort, painful neuroma, burning sensation, superficial hypersensitivity, no elbow extension deficit, or ulnar nerve subluxation. Sensory lost improved in all patients after the surgery and gradually improve after reevaluation at six-month subjective scale with good to excellent results. All preoperative electrophysiological studies were considered in all cases with abnormal results and with postoperative comparison which result findings five (83%) residual changes but one patient (16%) who has residual impaired because of the preoperative findings of evidence of axonal loss. This patient presented with severe preoperative compromise of the intrinsic musculature of the hand and subjective persistent numbness of the ulnar 1(1/2) side digits distribution, the postoperative NCV shows evidence of axonal loss but after 6 months postoperative NCS shows interval improvement with moderate prolonged. This patient improved the subjective parameters of modified bishop scoring system to good score result even when with persistent ulnar nerve distribution numbness and subjectively claimed that numbness decrease by 90% postoperatively, and objective parameter was satisfactory with overall clinical improvement. Overall, these six cases were good to excellent subjective improvement of the result and also objective parameters in grip strength improvement. Patients usually complain of sensory symptoms rather than muscle weakness so the result of the surgery was considered satisfactory by the patients, as their major complaints were related to the sensory symptoms.

 There were no complications in this series of six patients with good patient satisfaction and successful outcome without untoward complication.

## 5. Discussion

There is no gold standard in the surgical management of the cubital tunnel syndrome for the main reason that no single standard consensus of primary problem in nerve compression. In two groups of authors one believed that nerve compression is caused by overlying structure [12] and that the syndrome is best treated by decompression of the ulnar nerve without removing it from its bed. Other group of authors are citing evidence that the nerve is under tension with elbow flexion [13]. That can only be relieved by placing the nerve anterior to the medial epicondyle [14].

Various surgical techniques for decompression of the ulnar nerve have been described in the literature, and a definitive gold standard does not exist [9]. Heithoff (1999) stated that all surgical techniques for cubital tunnel syndrome yielded similar results and that the choice of surgical technique should be based on simplicity [15].

 The endoscopic approach to in situ decompression in our series of six patients has a rapidity of postoperative improvement of symptoms compatible to the study of Hoffmann and Siemionow in 2006. A completely new approach to surgery which enables to see and to do more through much smaller incision than those used by more traditional technique [16]. It is a minimally invasive alternative for decompression of the ulnar nerve at the elbow, aiming to minimize the trauma to the tissues and improved postoperative recovery to the patients. Its theoretical advantages over the classical open approach are the immediate well being of the patient, decreased invasiveness, minimal vascular complications, and less scar discomfort.

 In our view, like that patient with our inclusion criteria will be benefited from endoscopic release. A review with various authors (Assmus, 1994; Nathan et al., 1992, 1995; Pavellza et al., 2004; Taniguchi et al, 2002; Tsai et al., 1999) is that the transposition of the ulnar nerve is not only unnecessary for the treatment of cubital tunnel syndrome, but that it may often be harmful and seriously disadvantageous, considering its potential complications (Heithofff, 1999, Mariani et al., 1999) [16]. The efficacy of simple decompression for the treatment of cubital tunnel syndrome was first reported by Osborne (1957). Since then, many authors have reported good to excellent results with simple decompression (Chan et al., 1980). Adelaar et al., (1984), Bismmler and Meyer (1996), Davies et al., (1991), and Foster and Edshage (1981) compared simple decompression with anterior transposition, and found no significant difference in clinical outcome [15]. Prospective randomized studies have shown results of simple decompression to be equal to those of anterior transposition [17, 18]. In situ decompression also appears to have a low failure rate. But open decompression techniques require a longer incision, involve massive soft tissue dissection and are common associated with large postoperative scarring and wound tenderness.

 We can conclude that anterior transposition versus simple decompression has no significant difference in clinical outcome. A recent comparison between endoscopic techniques and in situ decompression demonstrated statistically significant less pain and greater satisfaction with the endoscopic technique [9]. By minimally invasive with a direct visualisation to the ulnar nerve by endoscopic guidance can be visualised better more the potential compression sites of ulnar nerve entrapment, and all potential sites of nerve compression in the elbow region were released without damage to the macroscopically visible nerves. With endoscopy, a long portion of the nerve can be released without damage to cutaneous innervation. Limited soft-tissue dissection with the preservation of the anatomy, especially vascularisation, minimises perineural fibrosis and enables rapid postoperative rehabilitation and can be safe and reliable with good functional and aesthetic result. According to the study by Hoffmann and Siemionow, the results of endoscopic release showed better functional recovery, lower morbidity and faster return to manual labour compared to conventional open method, and there were no serious complications.

We conclude that endoscopic release is a safe procedure in the hands of the experienced surgeon with careful protection of the nerve and the branches and a complete decompression. The observed postoperative results demonstrated that this surgical technique to the ulnar nerve at the level of the elbow was very effective, and there was improvement in the clinical and electrophysiological outcomes in all the subjects who underwent the procedure. This procedure is a relatively alternative to the conventional open release technique in the uncomplicated cases. The short term has proven to be a safe and effective tool for the operative management of uncomplicated cases. The results showed better immediate functional recovery, lower morbidity, and faster and shorter rehabilitation time, and return to active activity was rapid or quicker return of the patients to their daily activity, acceptable aesthetic result and above all with a high patient satisfaction rate. Endoscopic cubital tunnel release theoretically has better short-term outcome comparing to other technique in decompression but, however, to date, the number of studies reporting the case remains small.

## Figures and Tables

**Table 1:** The profile of the patient.

Case	Age/Sex	Job	Dexterity	Limb affected
1	33 F	Dental hygienist	R	L
2	52 F	Office assistant	R	R
3	58 M	Table task work	Ambidexterity	R
4	64 M	Home	R	R
5	77 M	Home	R	L
6	63 M	Home	R	R

R = right.

L = left.

**Table 2:** Dellon's stages of the ulnar nerve compression at the elbow.

Case	Sensory			Motors			Tests		
	Mild	Moderate	Severe	Mild	Moderate	Severe	Mild	Moderate	Severe

1		+			+			+	
2		+			+			+	
3		+			+			+	
4			+			+			+
5			+		+				+
6			+		+				+

+, positive.

**Table 3:** Modified Bishop's scoring system.

Case	Age	Severity of residual	Symptoms improvement	Work status	Strength	Sensibility
1	33	2	2	2	1	1
2	52	2	2	2	1	1
3	58	2	2	2	1	0
4	64	2	2	na	1	0
5	77	2	2	na	1	0
6	63	2	2	na	1	0

Score: 8–9 excellent; 5–7 good; 3–4 fair; 0–2 poor.

na. not applicable.

**Table 4:** Nerve conduction study.

Case	Age	Dellon's scale	Preoperative NCS		Postoperative NVS		Bishop scale
			Motor NC	Sensory and mixed NC	Motor NC	Sensory and mixed NC	
1	33	II	P	P	Mild to moderate P	Mild to moderate P	8
2	52	II	P	P	Improved	Improved	8
3	58	II	P	P	Mild to moderate P	Mild to moderate P	6
4	64	III	P	P	Improvement	Improvement	5
5	77	III	P	P	Normalized	Normalized	5
6	63	III	P	P	Mild to moderate P	Mild to moderate P	5

P = *prolonged.*
